# The role of forensic physicians in assessing cannabis detection test results in the trans community

**DOI:** 10.1177/00258024211057953

**Published:** 2021-11-11

**Authors:** Michael Ni’Man, Nikolas P Lemos

**Affiliations:** Cameron Forensic Medical Sciences, William Harvey Research Institute, Barts and The London School of Medicine and Dentistry, 324882 Queen Mary University of London, London, England, UK

As the usage of medicinal marijuana rises and cannabis decriminalisation becomes more accepted globally,^1^ it is crucial for clinical forensic medicine to equip itself to handle this future landscape. However, the limited knowledge base that exists on the gender differences in delta-9-tetrahydrocannabinol (THC) retention inhibits a forensic physician's ability to make robust assessments of cannabis detection test results.^2^ Furthermore, noticeably absent in any gender discussion is trans THC retention (only five results are returned for the PubMed search term “transgender THC,” none of which were related to retention). This letter seeks to draw on the current knowledge base in the cis-population to suggest how THC retention may occur in trans individuals.

It's held that average women have a higher percentage body fat than average men.^3^ Which, due to THC's lipophilic nature, results in women, on average, having a greater capacity for THC storage and consequently retention. Another study found that women have a greater cannabinoid receptor 1 (CB1, the receptor THC binds to) availability than men.^4^ Therefore, women have a greater opportunity to uptake and re-uptake THC (and its metabolites) in the body, retaining it for longer. On the other hand, female rodents have been shown to have a higher number of the liver enzymes that metabolise THC.^5^ Therefore, you might expect women to have greater THC metabolism and consequently faster elimination. Women are also more likely to suffer from stress-related disorders^6^ which would result in greater hypothalamic-pituitary adrenal (HPA) axis activation freeing more THC from fat cells.

Research on consumption found that men consume marijuana in greater amounts and at higher rates, meaning men have a greater concentration of THC to eliminate.^5^ Conversely, another study also describes how men are more likely to be resistant to antilipolytic effects.^3^ In other words, men are more likely to break down adipose and thus release THC.

Testosterone and progesterone have both been found to potentiate corticotrophin-releasing hormone (CRH) stimulated adrenocorticotropic hormone (ACTH) release.^7,8^ However, if we consider the effect THC has on each of these sex hormones, a difference emerges. Research has shown THC can cause a small rise in testosterone concentration which would therefore result in more HPA axis induction.^9^ Conversely, THC has been found to inhibit progesterone production, which would lead to less HPA axis activation.^10^

Drawing from the aforementioned evidence, trans-individuals may experience both stronger and weaker THC retention. Stronger THC retention may be seen in transwomen as a result of an increase in total body fat after hormonal transition, resulting in greater THC storage availability.^11^ Whilst, due to trans-individuals being more likely to suffer from stress-related disorders they may also experience weaker THC retention as a result of increased HPA axis induction.^12^ Furthermore, the THC interaction with sex hormones is especially pertinent as it relates to trans-individuals and the hormone therapy they may be taking.

As part of the regulations set out by The Faculty of Forensic & Legal Medicine of the Royal College of Physicians, forensic physicians are expected to interpret drug interactions, impairment, and intoxication in a variety of situations.^13^ With the 2014 amendment to the Road Traffic Act 1988 introducing legal limits to THC, and the increasing prevalence of trans-athletes, it is essential for clinical forensic medicine to understand the context of the results they see.^14^ It is not impossible to imagine a scenario where an athlete or driver fails a cannabis test despite being abstinent for days or weeks due to either taking part in a sporting event that activates the HPA axis or having strong THC retention factors in their physiological make-up.

Shifting societal context necessitates forensic physicians and forensic toxicologists to better understand the difference between sex and gender, as well as the retention differences in genders and the trans community. The points highlighted in this letter may help to better inform forensic practitioners (i.e., physicians and toxicologists) assessments of cannabis detection results in the trans population, based on the presence of Body Mass Index (BMI) >30, female gender (cis or trans), stress-related disorders, or on transgender hormonal therapy. Based on this, future researchers could explore whether these data points may offer an ‘at the point of investigation’ scoring system to assist forensic physicians and toxicologists. Moreover, the novelty of this domain and the current weak evidence base surrounding transgender cannabis use, should encourage greater toxicologic research to be performed.
